# Scaffold stability and P14′ residue steric hindrance in the differential inhibition of FXIIa by *Aedes aegypti* trypsin inhibitor versus Infestin-4

**DOI:** 10.1042/BSR20220421

**Published:** 2022-05-16

**Authors:** Varsha Ashok Walvekar, Karthik Ramesh, Muthu Kannan, R. Manjunatha Kini, J. Sivaraman, Yu Keung Mok

**Affiliations:** 1 Department of Biological Sciences, 16 Science Drive 4, National University of Singapore, Singapore 117558; 2 Department of Pharmacology, Yong Loo Lin School of Medicine, National University of Singapore, Singapore 117600

**Keywords:** Aedes aegypti trypsin inhibitor, Factor XIa, Infestin, Kazal-type protease inhibitor

## Abstract

Kazal-type protease inhibitors strictly regulate Factor XIIa (FXIIa), a blood-clotting serine protease. However, when negatively charged surface of prosthetic device come into contact with FXII, it undergoes conformational change and auto-activation, leading to thrombus formation. Some research suggests that Kazal-type protease inhibitor specificity against FXIIa is governed solely by the reactive-site loop sequence, as this sequence makes most-if not all-of the direct contacts with FXIIa. Here, we sought to compare the inhibitory properties of two Kazal-type inhibitors, Infestin-4 (Inf4), a potent inhibitor of FXIIa, and *Aedes aegypti* trypsin inhibitor (AaTI), which does not inhibit FXIIa, to better understand Kazal-type protease specificity and determine the structural components responsible for inhibition. There are only three residue differences in the reactive-site loop between AaTI and Inf4. Through site-directed mutagenesis, we show that the reactive-site loop is only partially responsible for the inhibitory specificity of these proteases. The protein scaffold of AaTI is unstable due to an elongated C5C6 region. Through chimeric study, we show that swapping the protease-binding loop and the C5C6 region from Inf4 with that of AaTI can partially enhance the inhibitory activity of the AaTI_Inf4 chimera. Furthermore, the additional substitution of Asn at the P14′ position of AaTI with Gly (Gly27 in Inf4) absolves the steric clashing between AaTI and the surface 140-loop of FXIIa, and increases the inhibition of the chimeric AaTI to match that of wild-type Inf4. Our findings suggest that ancillary regions in addition to the reactive-site loop sequence are important factors driving Kazal-type inhibitor specificity.

## Introduction

Factor XIIa (FXIIa), also known as Hageman factor, is a serine protease that plays a pivotal role in the intrinsic pathway of the blood coagulation cascade. Studies have reported that zymogen FXII circulates in the blood and becomes auto-activated upon exposure to negatively charged surfaces [1], through direct binding of domains rich in positively charged lysine residues and induced conformational change in FXII [2]. Upon activation, FXIIa stimulates FXI and initiates a series of proteolytic events leading to fibrin clot formation. This is particularly problematic in the medical field, as FXIIa can be auto-activated due to the presence of blood-contacting medical devices such as central venous catheters, prosthetic heart valves and intravenous cannulas [3]. This contact leads to thrombus formation and thus poses a serious problem in surgery. FXIIa also participates in chronic inflammatory diseases via activation of the kallikrein-kinin system and causes sepsis and hypotension in patients with bacterial infection [4]. Novel findings in FXIIa-deficient mice, which fail to present with bleeding disorders and are protected against thromboembolism [5], has rendered FXIIa an important target in the general medical field.

FXIIa, like other blood-clotting serine proteases, is under strict regulation by protease inhibitors. Kazal-type serine protease inhibitors are small inhibitors of 40–60 amino acids that contain the characteristic Kazal-like domain [6]. The Kazal inhibitor scaffold is maintained via three highly conserved disulfide bridges between cysteine residues: Cys-I and Cys-V, Cys-II and Cys-IV, and Cys-III and Cys-VI. A characteristic Kazal domain comprises one α-helix and a three-stranded β-sheet connected via peptide loops of variable lengths. The reactive-site loop or protease-binding loop comprises an extended convex-shaped loop harboring the P1 residue along with other primary and secondary contacts required for interaction with the cognate enzyme [7]. Kazal-type inhibitors are divided into classical and non-classical inhibitors based on the length of the spacer that separates Cys-I from Cys-II (*m*), and Cys-IV from Cys-V (*n*). Classical Kazal-type inhibitors are found with spacer lengths in the range *m* = 8–16 and *n* = 2, whereas non-classical Kazal-type inhibitors have *m* = 1–5 and *n* = 3–8 [8].

Infestin (Inf) is a Kazal-type inhibitor from the midgut of *Triatoma infestans*, an insect vector of Chagas’ disease [9]. Infestin has four domains, referred to as Inf1-4, but only domain 1 and 4 have been shown to have specific inhibitory activities and have thus been extensively studied: whereas Inf1 inhibits thrombin and trypsin, Inf4 inhibits FXIIa (*K*_i_ = 128 pM), plasmin (*K*_i_ = 2.1 nM) and FXa [10,11]. The crystal structures of Inf4 [11] and FXIIa [12] separately are available, as is the complex between Inf1 and trypsin [11]; however, there is no complex structure for Inf4 with FXIIa. Antithrombotic assessment in mice shows that Inf4 serves as a target for preventing surface- and FeCl_3_-induced arterial and venous thrombosis [13]. In addition, mice suffering from FXIIa-mediated cerebral ischemia show improved stroke outcomes and better neurological performance upon treatment with recombinant Inf4 [14]. Furthermore, off-target inhibition of FXa and plasmin by wild-type Inf4 can result in bleeding issues; these coagulation issues can be abated by treatment with Inf4 constructs bearing mutations in the reactive-site loop [15].

The Kazal-type inhibitor, *Aedes aegypti* trypsin inhibitor (AaTI), is a novel trypsin Kazal-type inhibitor from *Aedes aegypti*, the primary vector of mosquito-borne diseases like dengue [16,17]. AaTI is a competitive inhibitor of plasmin (*K*_i_ = 3.8 nM) but it does not inhibit kallikreins, FXa or FXIIa [17], and only weakly inhibits thrombin (*K*_i_ = 320 nM) [18]. AaTI has a similar structure to that of Inf4 [19], and with only a 3-residue difference in the reactive-site loop sequence. The reactive-site loop sequence makes most-if not all-of the direct contacts with the protease and is thought to be the sole factor that govern the inhibitor specificity. Inf4 and AaTI similarly inhibit plasmin but, despite the high structural and reactive-site loop similarity, AaTI cannot inhibit FXIIa. We previously showed that dengue virus captures plasmin to increase the permeability of the midgut barrier in the mosquito for infection, whereas AaTI inhibits plasmin and suppresses dengue virus infection of the mosquito [20]. We also previously determined the crystal structure of the AaTI:plasmin complex and found that, in addition to the reactive-site loop sequence, the reduced scaffold stability of AaTI, due to an elongated C5C6 region, leads to steric clashing that likely influences its potential to inhibit proteases [21].

In the present study, we compare the inhibition of FXIIa by Inf4 and AaTI and confirm that both the reactive-site loop sequence and scaffold stability of the inhibitor are essential for serine protease inhibition by Kazal-type inhibitors. In addition, we show that an Asn residue at the P14′ position of AaTI is partially responsible for its lack of inhibition of FXIIa, likely causing a clash with the surface 140-loop of FXIIa.

## Materials and methods

### Recombinant protein production of AaTI and inf4

Bacterial codon optimized genes encoding for AaTI and Inf4 were purchased from Bio Basic Asia Pacific Pte Ltd (Singapore) and subcloned into a modified version of the pET32a (Merck; Kenilworth, NJ) prokaryotic expression vector. Protein expression was carried out using IPTG (isopropyl β-d-1-thiogalactopyranoside) induction in SHuffle T7 competent *Escherichia coli* cells (New England Biolabs; Ipswich, MA). His-tagged proteins were purified using the cOmplete His-tag purification resin (Merck) and the eluate was further purified using size-exclusion chromatography using Hiload 16/600 Superdex 75 pg column (16 × 600 mm) (GE Healthcare; Chicago, IL). The His-tag remains intact in all the recombinant wild-type and chimeric proteins used in this study. Purified proteins were tested for inhibitory activities against human FXIIa (Merck). All wild-type, mutant and chimeric proteins used in this work were dissolved in a 50 mM Tris-HCl, pH 7.4 buffer, with 200 mM NaCl.

### Preparation of chimera proteins

Chimeric proteins were designed via site-directed mutagenesis with the forward and reverse primers incorporating the desired mutation. For the insertion of longer fragments, half of the desired mutations were incorporated into the 5′ ends of the forward and reverse primers such that the primers annealed back-to-back. PCR amplification of the plasmids was performed using the Q5 Hot Start High-Fidelity 2× Master Mix (New England Biolabs). Linear PCR products were incubated with T4 Polynucleotide Kinase (New England Biolabs) and ligated with T4 DNA ligase (New England Biolabs). Amplified plasmids were subjected to digestion with DpnI (New England Biolabs) enzyme at 37°C for 1 h and transformed into chemically competent DH5α cells. Plasmid DNA was isolated from single colonies and sequenced to confirm the desired mutations. Confirmed plasmids were transformed into T7 SHuffle competent *E. coli* cells for expression and purification. The elution profiles of all chimeras show proper folding of all of the proteins (Supplementary Figure S1).

### Chromogenic assays for protease inhibition

Inhibitory activities of AaTI and Inf4 on human FXIIa (Merck) were assessed via chromogenic assays using the Chromogenix S-2302 chromogenic substrate (Diapharma; West Chester, OH). For IC_50_ determination, FXIIa was pre-incubated with different concentrations of inhibitors. The generation of p-nitroaniline (pNA) was determined by measuring the absorbance at 405 nm using a absorbance plate reader (Tecan; Männedorf, Switzerland). The reaction mixture consisted of 40 µl FXIIa (10 nM), 40 µl inhibitor, and 40 µl chromogenic substrate (1 mM). All reaction components were prepared in 50 mM Tris pH 7.4 containing 100 mM NaCl.

### Molecular dynamics simulation

Docked models were generated using the ClusPro protein-protein docking webserver [22–24]. Docked models were selected based on the typical interaction of Kazal-type inhibitors with their cognate enzymes, such that the P1 Arg of Inf4 inserted into the S1 pocket of FXIIa. MD simulation was performed on the docked model using GROMACS 5.1.1 package with Amber ff99SB-ILDN as the force field [25,26]. The system was solvated using extended simple point charge (SPC216) in a triclinic-shaped box. The net charge of the system was neutralized by adding counterions, such as Cl^−^ and Na^+^. Particle Mesh Ewald electrostatics [27] was used for calculations of electrostatic and van der Waals interactions with a cut-off of 1.0 nm. Steepest descent energy minimization was performed until a force convergence of 1000.0 kJ/mol/nm was achieved. The LINCS algorithm was used to constrain all bond angles [28]. An equilibration of 0.1 ns was performed under NVT conditions (constant number of particles, volume and temperature); a constant temperature of 300 K was confirmed using the V-rescale thermostat. Equilibration of 0.1 ns was also performed under NPT conditions (constant number of particles, pressure and temperature) to maintain the pressure at 1.0 bar using Parrinello-Rahman [29]. MD simulation was run for 100 ns and trajectory frames were saved every 10 ps. The stability of the Inf4:FXIIa complex was checked by the RMSD and RMSF plots to be acceptable (Supplementary Figure S2).

### The PCA and FEL analysis

Principal component analysis (PCA) of trajectories was carried out to reveal the atomic fluctuations using eigenvectors of the covariance matrix. The cosine content (ci) of each principal component (Pi) of the covariance matrix was calculated to represent the free energy landscape defined by PCA analysis. The values of the cosine content ranged between 0 (no cosine) and 1 (perfect cosine) over the total simulation period (t), where

ci=2Τ(∫cos(iπt)pi(t)dt)2(∫pi(t)2dt)−1

Studies have shown that cosine values of 0.2 or lower that correspond to the first two principal components (PC) can yield qualitatively related and better results. Therefore, the first 20 PCs were generated and analyzed based on their cosine values. The first two PCs with scores lower than 0.2 were used for free-energy landscape (FEL) generation, resulting in clusters of protein–protein complexes based on their energy levels; these clusters aid in identifying the best energy conformations of the investigated complexes [30].

### Quantification and statistical analysis

For IC_50_ calculations, log[inhibitor concentration] vs. percentage normalized inhibition was plotted using a non-linear regression curve function, created with Kaleidagraph 4.5.2 (Synergy Software; Reading, PA). All data represent the mean ± SD for triplicate values. Table 1 shows the IC_50_ values of all Inf4 and AaTI mutants and chimeras used in the present study toward FXIIa.

**Table 1:** IC_50_ values of Inf4 and AaTI mutants and chimeras toward FXIIa

	IC_50_ values toward FXIIa (nM)
Inf4	
Wild-type Inf4	3.1 ± 0.5
Inf4_F10P	11.6 ± 1.1
Inf4_N12I	8.4 ± 0.9
Inf4_V14M	9.9 ± 1.6
Inf4_F10P+N12I	22.8 ± 4.7
Inf4_N12I+V14M	7.9 ± 2.1
Inf4_F10P+V14M	42.1 ± 8.4
Inf4_F10P+N12I+V14M	70.5 ± 9.7
Inf4_AaTI C3C4	63.6 ± 13.8
Inf4_AaTI C4C5	8.9 ± 2.0
Inf4_AaTI C5C6	28.2 ± 7.3
Inf4_AaTI C6	6.2 ± 0.8
Inf4_G25N	54.1 ± 21.8
**AaTI**	
Wild-type AaTI	N.A.
AaTI_Inf4 C1C3	4680 ± 3834.6
AaTI_Inf4 C1C3+C3C4	146.9 ± 42.9
AaTI_Inf4 C1C3+C5C6	112.6 ± 16.7
AaTI_Inf4 C1C3+F10A	610.3 ± 205.9
AaTI_Inf4 C1C3+N27G	58.0 ± 14.8
AaTI_Inf4 C1C3+C5C6+N27G	11.4 ± 3.1

## Results

### The reactive-site loop sequence of AaTI is only partially responsible for its loss of inhibition toward FXIIa

**Figure 1:**
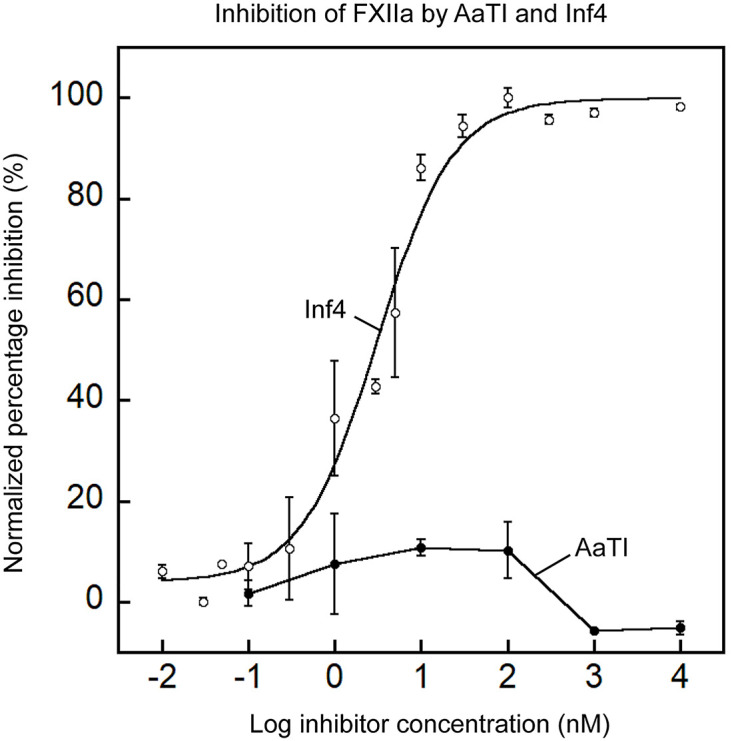
Inhibitory activity of Inf4 and AaTI toward FXIIa Plots showing the inhibitory activity of wild-type AaTI (closed circles) and Inf4 (open circles) on FXIIa. The IC_50_ values for Inf4 inhibition against FXIIa (derived from curve fitting) is 3.1 ± 0.5 nM. AaTI did not display any detectable inhibition toward FXIIa. Data represent the mean ± SEM for triplicate values.

**Figure 2:**
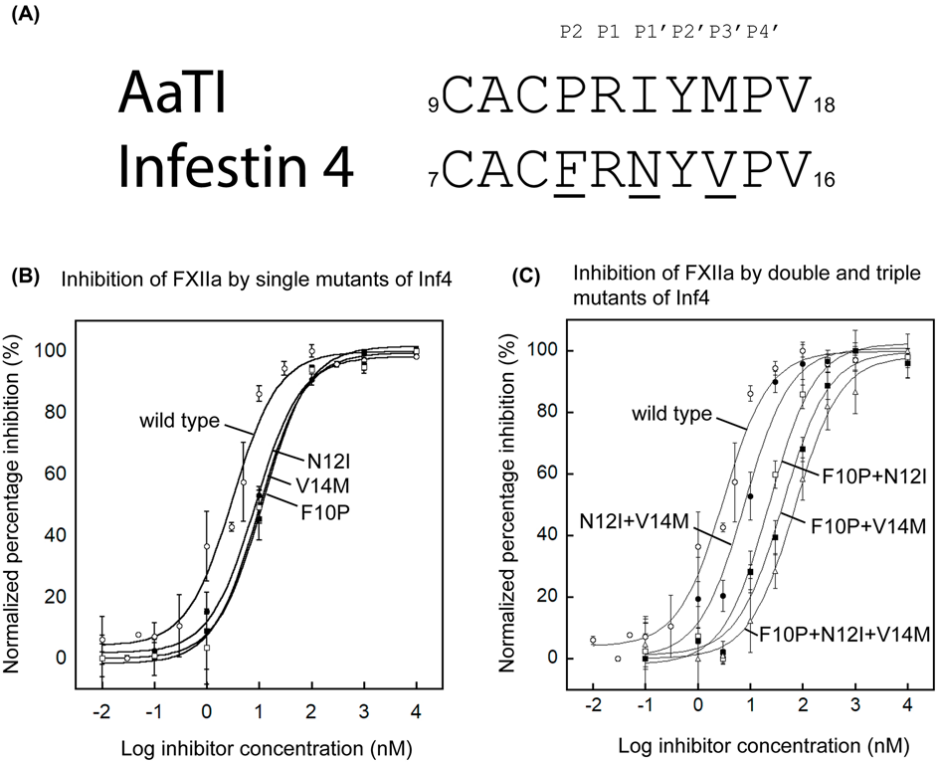
Inhibitory activities of wild-type and mutants of Inf4 on FXIIa activity (**A**) Sequence alignment of the reactive-site loops of AaTI and Inf4 from residues P5 to P5′. Residues in Inf4 that are different from those in AaTI are underlined. Mutations to these residues were made in Inf4 to determine the relative contributions of these residues in prohibiting the activity of AaTI. (**B**) Plots showing the relative inhibition of FXIIa by wild-type Inf4 (open circles), Inf4_F10P (closed squares), Inf4_N12I (closed circles) and Inf4_V14M (open squares). The IC_50_ values derived from curve fittings are 3.1 ± 0.5 nM for wild-type Inf4, 11.6 ± 1.1 nM for Inf4_F10P, 8.4 ± 0.9 nM for Inf4_N12I and 9.9 ± 1.6 nM for Inf4_V14M. Data represent the mean ± SEM for triplicate values. (**C**) Plots showing the relative inhibition of FXIIa by wild-type Inf4 (open circles), Inf4_N12I+V14M (closed circles), Inf4_F10P+N12I (open squares), Inf4_F10P+V14M (closed squares) and Inf4_F10P+N12I+V14M (open triangles) on FXIIa. The IC_50_ values derived from curve fittings are 3.1 ± 0.5 nM for wild-type Inf4, 7.9 ± 2.1 nM for Inf4_N12I+V14M, 22.8 ± 4.7 nM for Inf4_F10P+N12I, 42.1 ± 8.4 nM for Inf4_F10P+V14M and 70.5 ± 9.7 nM for Inf4_F10P+N12I+V14M. Data represent the mean ± SEM for triplicate values.

We first assessed the inhibitory activities of AaTI and Inf4 on human FXIIa using a chromogenic assay. IC_50_ values, instead of *K*i values, are chosen for quantitative comparison of changes in inhibitory activity instead of binding affinity between wild-type and mutant proteins. Inf4 displayed potent inhibitory activity against FXIIa with an IC_50_ value of 3.1 ± 0.5 nM. In contrast, AaTI did not show any significant inhibition of FXIIa up to 10 μM (Figure 1). Previous work suggests that most, if not all, of the direct contact between the inhibitors and the protease involves the reactive-site loop [21]. Thus, we compared the sequences of the reactive-site loops of Inf4 and AaTI from P5 to P5′ residues and found a difference of only three residues at positions P2, P1′ and P3′ (Figure 2A). To determine if this difference in the reactive-site loop sequence contributes to the much higher inhibition by Inf4 toward FXIIa, we mutated each of these residues on Inf4 to the corresponding residues in AaTI separately and assessed for changes in inhibitory activity. None of the three single mutants caused a significant loss in inhibition towards FXIIa, with all three mutants showing a similar behaviour in terms of inhibitory potential (Figure 2B).

To determine whether a combination of these mutations would alter inhibition, we generated double and triple mutants of Inf4 at the reactive-site loop. The double mutant Inf4_N12I+V14M showed a similar level of reduction in inhibition towards FXIIa as the single mutants. However, the Inf4_F10P+N12I and Inf4_F10P+V14M double mutants showed quite significant reductions in inhibitory potential, with IC_50_ values of 22.8 ± 4.7 and 42.1 ± 8.4 nM, respectively. This suggests that the P2 residue, Phe10, could play a more significant role in the reactive-site loop for the inhibition of FXIIa. Furthermore, the triple mutant, Inf4_F10P+N12I+V14M led to an even greater loss in inhibitory activity toward FXIIa, with an IC_50_ value of 70.5 ± 9.7 nM (Figure 2C). However, the activity of this triple mutant-now bearing the same reactive-site loop sequence as AaTI-still has a much higher inhibitory potential than wild-type AaTI. These results suggest that the differences in the reactive-site loop of AaTI only partially contribute to its lack of inhibition towards FXIIa.

### Both the C3C4 and C5C6 regions of AaTI also contribute to its loss of inhibition on FXIIa

**Figure 3:**
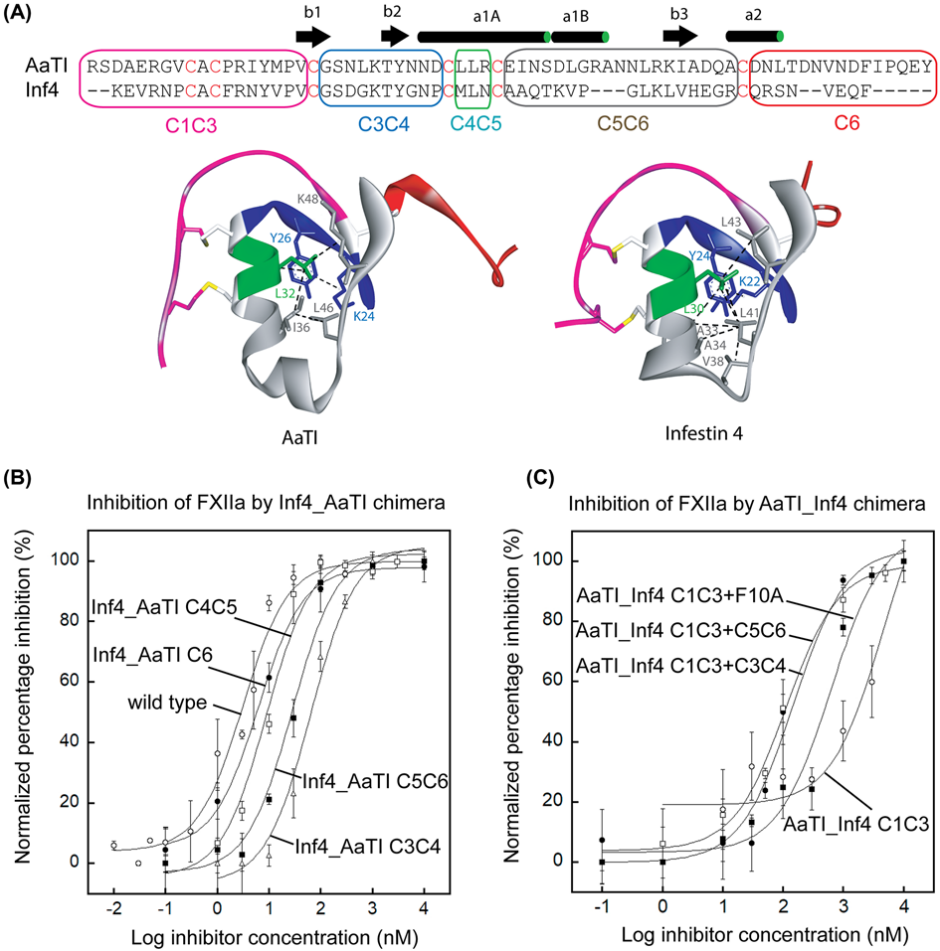
Inhibitory activities of Inf4 and AaTI chimera on FXIIa (**A**) Sequence alignment of AaTI and Inf4 showing an elongated region between Cys V and Cys VI in AaTI. Chimeras were designed with different substitutions: N-terminus to Cys III (C1C3, magenta box), Cys III to Cys IV (C3C4, blue box), Cys IV to Cys V (C4C5, green box), Cys V to Cys VI (C5C6, grey box), and Cys VI to C-terminus (C6, red box). Cys residues are highlighted in red. Secondary structures of AaTI are shown above the sequences. Ribbon diagrams below the sequences show the crystal structure of AaTI and the molecular model of Inf4 colored according to the scheme above. All hydrophobic interactions in the inhibitor core are depicted as black dashed lines. Hydrophobic interactions, such as Pi-Pi, alkyl-alkyl, and Pi-alkyl interactions, were calculated by Discovery Studio Visualizer software using default parameters (6.0 Ǻ distance for Pi-Pi and 5.5 Ǻ for alkyl interactions). Residues involved in the hydrophobic core are shown as stick models and labeled. (**B**) Plots showing inhibition of FXIIa by wild-type Inf4 (open circles), Inf4_AaTI C3C4 (open triangles), Inf4_AaTI C4C5 (open squares), Inf4_AaTI C5C6 (closed squares) and Inf4_AaTI C6 (closed circles). The IC_50_ values derived from curve fittings are 3.1 ± 0.5 nM for wild-type Inf4, 63.6 ± 13.8 nM for Inf4_AaTI C3C4, 8.9 ± 2.0 nM for Inf4_AaTI C4C5, 28.2 ± 7.3 nM for Inf4_AaTI C5C6, and 6.2 ± 0.8 nM for Inf4_AaTI C6. Data represent the mean ± SEM for triplicate values. (**C**) Plots showing inhibition of FXIIa by AaTI_Inf4 C1C3 (open circles), AaTI_Inf4 C1C3+F10A (closed squares), AaTI_Inf4 C1C3+C3C4 (closed circles), and AaTI_Inf4 C1C3+C5C6 (open squares). The IC_50_ values derived from curve fittings are 4680.6 ± 3834.6 nM for AaTI_Inf4 C1C3, 610.3 ± 205.9 nM for AaTI_Inf4 C1C3+F10A, 146.9 ± 42.9 nM for AaTI_Inf4 C1C3+C3C4, and 112.6 ± 16.7 nM for AaTI_Inf4 C1C3+C5C6. Data represent the mean ± SEM for triplicate values.

We next sought to examine which other regions outside the reactive-site loop may be responsible for the loss of inhibition. We have previously shown that the elongated C5C6 region of AaTI (Figure 3A) contributes to an unstable protein scaffold based on GdnCl denaturation monitored by FarUV-CD (Supplementary Figure S3) [21]. The unstable protein scaffold may cause steric clashing between bulky residues in the reactive-site loop and the protease, leading to loss of plasmin inhibition [21]. Kazal-type inhibitors possess six Cys residues and three disulfide bonds, with the protein able to be divided into five regions: C1C3 (containing the N-terminal segment and the reactive-site loop), C3C4, C4C5, C5C6 and C6 (containing the C-terminal segment) (Figure 3A). Thus, we generated chimeras of Inf4 that contain different regions of AaTI and measured their degree of inhibition on FXIIa; of note, ‘Inf4_AaTI C3C4’ nomenclature describes a chimera of Inf4 with the C3C4 region replaced with that of AaTI.

Using these Inf4_AaTI chimeras, we found that replacement of either the C4C5 (IC_50_ = 8.9 ± 2.0 nM) or C6 (IC_50_ = 6.2 ± 0.8 nM) regions with those regions of AaTI caused only a slight reduction in the inhibitory activity of Inf4 on FXIIa (Figure 3B). However, substituting the C3C4 (IC_50_ = 63.6 ± 13.8 nM) and C5C6 (IC_50_ = 28.2 ± 7.3 nM) regions of AaTI caused profound drops in inhibitory activity; this was particularly evident for Inf4_AaTI C3C4, which showed an activity similar to that of the reactive-site loop triple mutant (IC_50_ = 70.5 ± 9.7 nM) (Figures 2C and 3B). We also tested the ‘reverse’ AaTI_Inf4 chimeras (following the same nomenclature). The AaTI_Inf4 C1C3 chimera, which contains the N-terminal segment and the whole reactive-site loop from Inf4, has a much better inhibitory activity toward FXIIa (IC_50_ = 4680 ± 3834.6 nM) than wild-type AaTI; albeit the activity does not compare with that of wild-type Inf4 (IC_50_ = 3.1 ± 0.5 nM) (Figure 3C). Together, these findings suggest that replacing the reactive-site loop and N-terminal segment with those of Inf4 also only partially rescues the inhibitory activity of AaTI toward FXIIa.

Finally, we tested a combined replacement, whereby we replaced the C1C3 region of Inf4 along with either the C3C4 or C5C6 regions of Inf4 in AaTI. Both of these chimeras showed significant recovery of AaTI inhibition on FXIIa, with IC_50_ values of 63.6 ± 13.8 nM for AaTI_Inf4 C1C3+C3C4 and 28.2 ± 7.3 nM for AaTI_Inf4 C1C3+C5C6 (Figure 3C). The low inhibitory activity of AaTI_Inf4 C1C3 is partly due to the unstable scaffold in AaTI, which causes the bulky residue in the reactive-site loop of the chimera to clash with the protease. By mutating Phe10 to Ala, the chimera AaTI_Inf4 C1C3+F10A has significant recovery in inhibition activity towards FXIIa (IC_50_ = 610.3 ± 205.9 nM) (Figure 3C). Of note, the recovery gained by removing the clashing Phe10 residue is not comparable with that of replacing the whole C5C6 region to stabilize the scaffold, as in AaTI_Inf4 C1C3+C5C6 (IC_50_ = 28.2 ± 7.3 nM), suggesting that the steric clash may come from residues in regions other than the reactive-site loop.

### Molecular modeling suggests steric clashing by P14′ Asn27 residue in the C3C4 region of AaTI with the 140-loop of FXIIa

**Figure 4:**
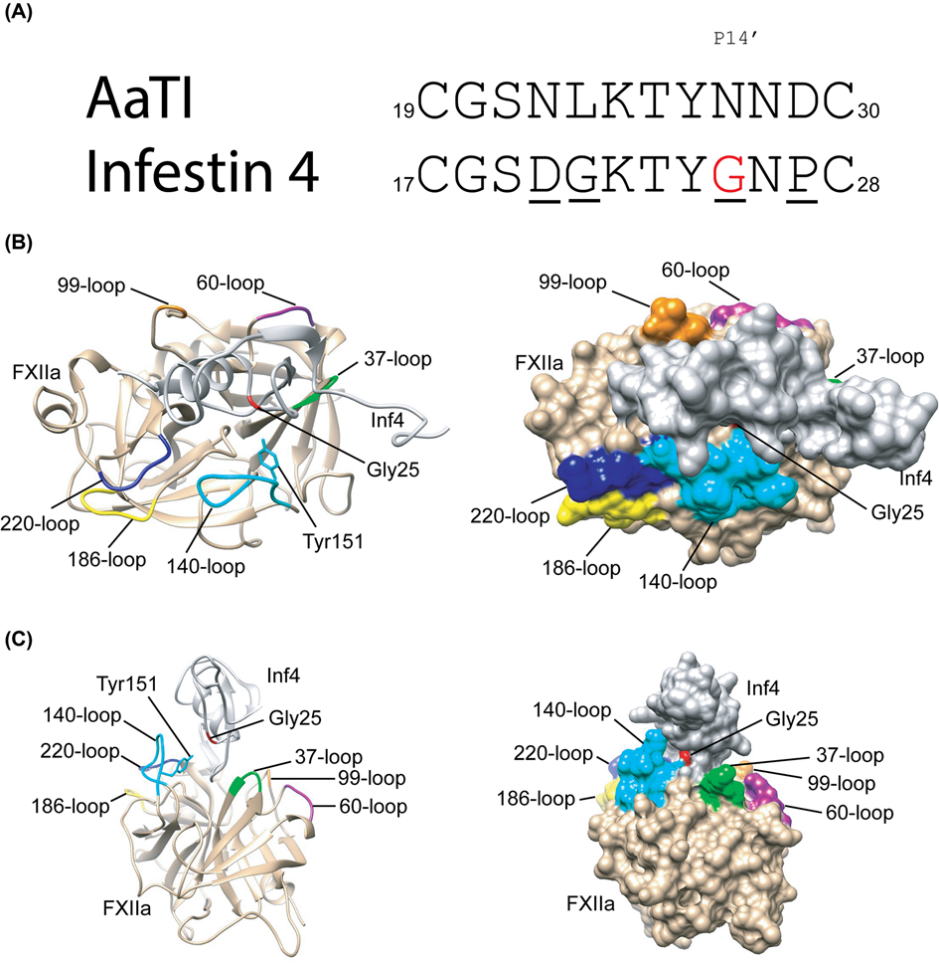
Ribbon and surface diagrams of the Inf4-FXIIa complex model (**A**) Sequence alignment of the C3C4 region comparing wild-type Inf4 and AaTI. The four different residues are underlined. The P14′ residue, Gly25, on Inf4 is colored red. (**B**) Ribbon and the corresponding surface diagram of the model complex structure between Inf4 (gray) and FXIIa (beige) showing the surface loops of FXIIa: 37-loop (green), 60-loop (purple), 99-loop (orange), 140-loop (cyan), 186-loop (yellow) and 220-loop (blue) as stated in Dementiev et al*.* (2018). The Gly25 (P14′) residue of Inf4 is shown in red color and the Tyr151 residue of FXIIa is shown as a cyan stick model. (**C**) A different view of the ribbon and corresponding surface diagram of the model of the Inf4-FXIIa complex. The color scheme is as described in (B). The diagrams are generated by the program UCSF Chimera.

As the C3C4 region is also responsible for the loss of inhibition in AaTI toward FXIIa (Figure 3C), we compared the sequences between Inf4 and AaTI in this region and found a difference of just four residues (Figure 4A). BLAST results for the Inf4 sequence indicates that the P14′ position is often occupied by small amino acid residues such as glycine, alanine or serine (Supplementary Figure S4). Indeed, Inf4 has a Gly25 residue in this position whereas the P14′ position in AaTI is occupied by Asn27 (Figure 4A). Using molecular dynamic simulation, we generated an *in silico* molecular model of the complex between Inf4 and FXIIa. The model suggested that most of the direct interactions between the two proteins occurs via the reactive-site loop of Inf4. However, residue Gly25 at P14′ of Inf4 lies close to residue Tyr151 on the surface 140-loop of FXIIa (Figure 4B,C). Replacement of Gly25 to an Asn residue as in AaTI could cause steric clashing between the inhibitor and the protease, especially if the scaffold is unstable. The other residues in the C3C4 region of Inf4 that are different from AaTI are Asp20, Gly21 and Pro27; however, these residues are not close to the FXIIa interaction.

### Three substitutions required to restore AaTI inhibition of FXIIa

**Figure 5:**
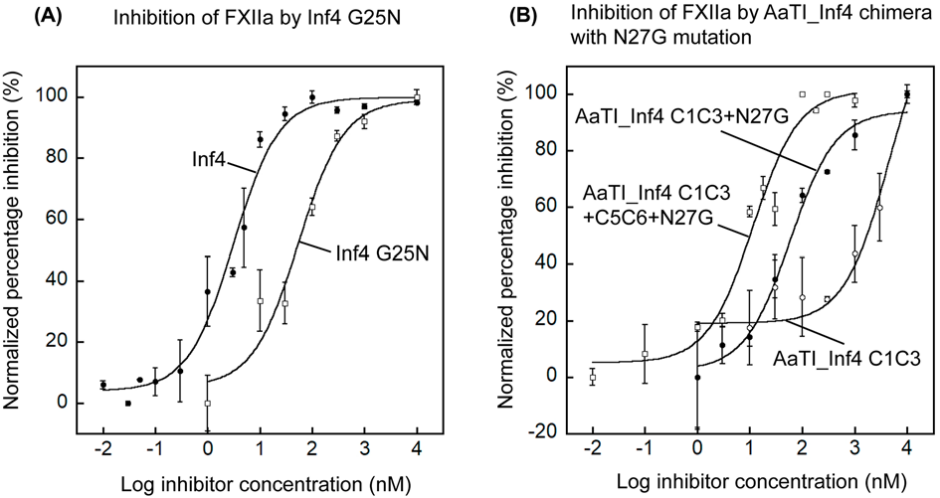
Inhibitory activities of Inf4 and AaTI_Inf4 C1C3 chimera with mutation at P14′ on FXIIa (**A**) Plots showing inhibition of FXIIa by wild-type Inf4 (closed circles) and Inf4_G25N mutant (open squares). The IC_50_ values derived from curve fittings are 3.1 ± 0.5 nM for wild-type Inf4 and 54.1 ± 21.8 nM for Inf4_G25N on FXIIa. Data represent the mean ± SEM for triplicate values. (**B**) Plots showing inhibition of FXIIa by AaTI_Inf4 C1C3 (open circles), AaTI_Inf4 C1C3+N27G (closed circles) and AaTI_Inf4 C1C3+C5C6+N27G (open squares). The IC_50_ value derived from curve fittings are 4680.6 ± 3834.6 nM for AaTI_Inf4 C1C3, 58.0 ± 14.8 nM for AaTI_Inf4 C1C3+N27G, and 11.4 ± 3.1 nM for AaTI_Inf4 C1C3+C5C6+N27G on FXIIa. Data represent the mean ± SEM for triplicate values.

To test the modeling results, we mutated the P14′ Gly25 residue in Inf4 to Asn. The inhibitory activity of the Inf4_G25N mutant was reduced significantly to an IC_50_ value of 54.1 ± 21.8 nM toward FXIIa from 3.1 ± 0.5 nM of Inf4 (Figure 5A). This suggest that an Asn residue at the P14′ position could indeed cause steric clashing with the 140-loop of FXIIa and lead to a reduced inhibition.

The AaTI chimera that contains the reactive-site loop from Inf4, AaTI_Inf4 C1C3, inhibits FXIIa only slightly better than the wild-type AaTI (Figure 5B). We thus mutated residue Asn27 to Gly in this chimera (AaTI_Inf4 C1C3+N27G) and found an improvement in the inhibition potential on FXIIa, with an IC_50_ value of 58.0 ± 14.8 nM (Figure 5B). The inhibitory activity of this chimera is better than that observed with the AaTI_Inf4 C1C3+C3C4 chimera (IC_50_ = 146.9 ± 42.9 nM), suggesting that most of the steric clashing from the C3C4 region is contributed by Asn27. Finally, the inclusion of a stable scaffold (C5C6 region) from Inf4 rendered the AaTI chimera (AaTI_Inf4 C1C3+C5C6+N27G) a potent inhibitor of FXIIa, with an IC_50_ value (11.4 ± 3.1 nM) very close to that of wild-type Inf4 (IC_50_ = 3.1 ± 0.5 nM) (Figure 5B).

## Discussion

All available crystal structures of Kazal-type inhibitors in complex with a protease show that the reactive-site loop makes most-if not all-of the direct contacts with the protease [21]. Indeed, based on the Inf4:FXIIa complex model, the reactive-site loop of Inf4 from residues Ala8 to Val14 (P4 to P3′ and sequence ‘^8^ACFRNYV^14^’) inserts into the catalytic groove of FXIIa and establishes direct close contacts with various subsites of the enzyme. The well-conserved P1 residue, Arg11, in Inf4 makes extensive interactions with FXIIa by extending deep inside the S1 subsite pocket of the protease and establishing salt-bridge interactions with Asp189 within the extremity of the pocket. In contrast, the P2 residue, Phe10, extends outwards from the S2 pocket; this observation is attributed to the fact that a Tyr residue (Tyr99) from the ‘99-loop’ occupies the S2 pocket in FXIIa, making the S2 subsite narrow and shielded from the P2 residue. Previously, we showed that the P2 Phe residue of Inf4 is essential for the inhibition of plasmin due to a ‘94-shunt’ and thus exposure of the S2 subsite, which is filled by the P2 Phe10 residue of Inf4 during binding [21]. The absence of the ‘94-shunt’ in FXIIa explains why mutating the P2 Phe residue to Pro does not cause a significant drop in the inhibitory activity of Inf4 on FXIIa. It also emphasizes the importance of surface loops in determining the specificity of Kazal-type inhibitors.

If we were to assume that the reactive site loop was the only direct point of contact between the inhibitor and the protease, additivity-based principles could be used to design a novel inhibitor based on sequence. As per the Laskowski mechanism of canonical inhibition [31], the inhibitory function of an inhibitor is coded into the protease binding loop region. Ideally, one should observe a gain in inhibitory function for an AaTI_Inf4 C1C3 chimera, in which the reactive-site loop of AaTI is swapped with that of Inf4. However, we observed only slight inhibition towards FXIIa for this chimera (Figure 3C); this further confirms that the binding of the inhibitor to the protease is not solely determined by the protease binding-loop sequence, but that other regions contribute to the inhibitory activity. This agrees with the conclusions of others that the protease binding loop region alone does not confer inhibition outcome by Kazal-type inhibitors, and that other scaffolding regions act as auxiliary elements [32].

We previously compared the inhibition of plasmin by AaTI, Inf4 and Inf1, and showed that, in addition to the reactive-site loop sequence, the stability of the scaffold plays an important role in Kazal-type inhibitor specificity [21]. We showed that an elongated C5C6 region in AaTI renders the hydrophobic core less compact and causes reduced scaffold stability. This instability, in turn, causes the bulky P2 residue to clash upon interaction with plasmin, leading to significantly reduced inhibition of plasmin by the AaTI_Inf4 C1C3 chimera [21]. Here, too, we show that a stable scaffold is important for the proper inhibition of FXIIa by Inf4; albeit, the surface loops between FXIIa and plasmin are different. These findings are consistent with the report by Marijanovic et al*.* that the specificity of serpin is not dictated only by the reactive centre loop sequence, but also by conformational dynamics and protein stability [33].

We show that the P14′ residue is an important determinant in FXIIa inhibition by Kazal-type inhibitors. Whereas this P14′ position is usually occupied by small amino acid residues such as Gly, Ala and Ser in most Kazal-type inhibitors, it is replaced by an Asn residue in AaTI (Supplementary Figure S4). Mutation of the P14′ Gly residue in Inf4 to Asn caused a significant 15-fold reduction in its inhibitory activity towards FXIIa. Despite similar structural topology of the chymo(trypsin)-like β-barrel architecture, serine proteases display differential enzyme specificity and selectivity, mainly attributed to variability in length and sequence of eight surface loops in proximity of the active site cleft [34]. The ‘140-loop’ or ‘autolysis-loop’ in FXIIa has a distinct conformation and harbours a characteristic Tyr151 residue, which is not found in other blood-coagulating serine proteases, such as plasmin, thrombin and FXa, but is found in trypsin (Supplementary Figure S5). The absence of Tyr151 in the 140-loop of plasmin is likely one of the reasons that AaTI can inhibit plasmin but not FXIIa. Steric clashing of this Tyr151 with amino acid residues bearing longer and branched side-chains at the P14′ position, such as Asn in AaTI, could prevent the binding of the inhibitor. We further speculate that this bulkier Tyr151 residue from the 140-loop of FXIIa might also clash with the P4′ Arg residue in Infestin-1 (Inf1), and may explain why Inf1 does not inhibit FXIIa [11]. Further investigations are needed to confirm this hypothesis.

The Tyr residues in the 140-loop of trypsin do not always cause steric clashing but may instead enhance and stabilize binding of the inhibitor to the enzyme. For example, in the Inf1:trypsin complex, the active-site cleft of trypsin is narrowed due to the presence of a bulky Tyr residue in the 140-loop. The Ser24 residue at the P14′ position of Inf1 fits well into this active-site groove and establishes hydrogen-bonding interactions with the Tyr residue [11]. AaTI is also a potent inhibitor of trypsin [17], which may suggest that the Tyr residue on the 140-loop of a different serine protease could serve an entirely different role. Tyr is also required in stabilizing the 140-loop of other Kazal-type inhibitors. Indeed, human pancreatic secretory trypsin inhibitor (PSTI) establishes hydrogen-bonding to a Tyr residue on the 140-loop of chymotrypsin, anchoring the loop and reducing loop dynamics. Site-directed mutational studies that disrupted this hydrogen bond also reduced the inhibitory action of PSTI by 25-fold, highlighting the importance of secondary contacts in Kazal-type inhibition [35].

Overall, our results highlight two critical aspects of inhibition by Kazal-type inhibitors in addition to the reactive-site loop: (1) scaffold-dependent stabilization of the reactive-site loop; and (2) length and sequence of the protease surface loops to avoid steric clashes with the inhibitor. Only through three substitutions could we convert AaTI into a reasonable FXIIa inhibitor: we required substitution of the protease-binding loop and region C5C6 of Inf4 as well as an Asn to Gly mutation at position 27. These ancillary regions play essential scaffolding roles in dictating inhibitor specificity, in addition to the reactive-site loop.

## Supplementary Material

Supplementary Figures S1-S5

## Data Availability

All data and reagents are available from the authors upon request.

## Abbreviations

AaTI: *Aedes aegypti* trypsin inhibitor
BLAST: Basic Local Alignment Search Tool
C1C3: region of the protein from the N-terminus to the residue before Cys-III
C3C4: region of the protein between Cys-III and Cys-IV
C4C5: region of the protein between Cys-IV and Cys-V
C5C6: region of the protein between Cys-V and Cys-VI
C6: region of the protein from the residue after Cys-VI to the C-terminus
Cys-I to -VI: first to sixth cysteine residue from the N-terminus of the protein, respectively
FEL: free-energy landscape
FXa: Factor Xa
FXIIa: Factor XIIa
GdnCl: guanidine hydrochloride
GROMACS: GROningen MAchine for Chemical Simulation
IC_50_: half maximal inhibitory concentration
Inf4: Infestin-4
IPTG: isopropyl β-d-1-thiogalactopyranoside
*K*_i_: inhibitory constant
LINCS: linear constraint solver
*m*: number of residues between Cys-I and Cys-II
MD: molecular dynamics
*n*: number of residues between Cys-IV and Cys-V
PCA: principal component analysis
SEM: standard error of mean
