# Supplementary material for: Scaffold stability and P14′ residue steric hindrance in the differential inhibition of FXIIa by Aedes aegypti trypsin inhibitor versus Infestin-4
Source: Biosci Rep. 2022 May 16;42(5):BSR20220421. doi: 10.1042/BSR20220421 (PMC9112662; doi:10.1042/BSR20220421)
Supplement: Supplementary Figures S1-S5 [file BSR-2022-0421_supp.pdf]

(A)

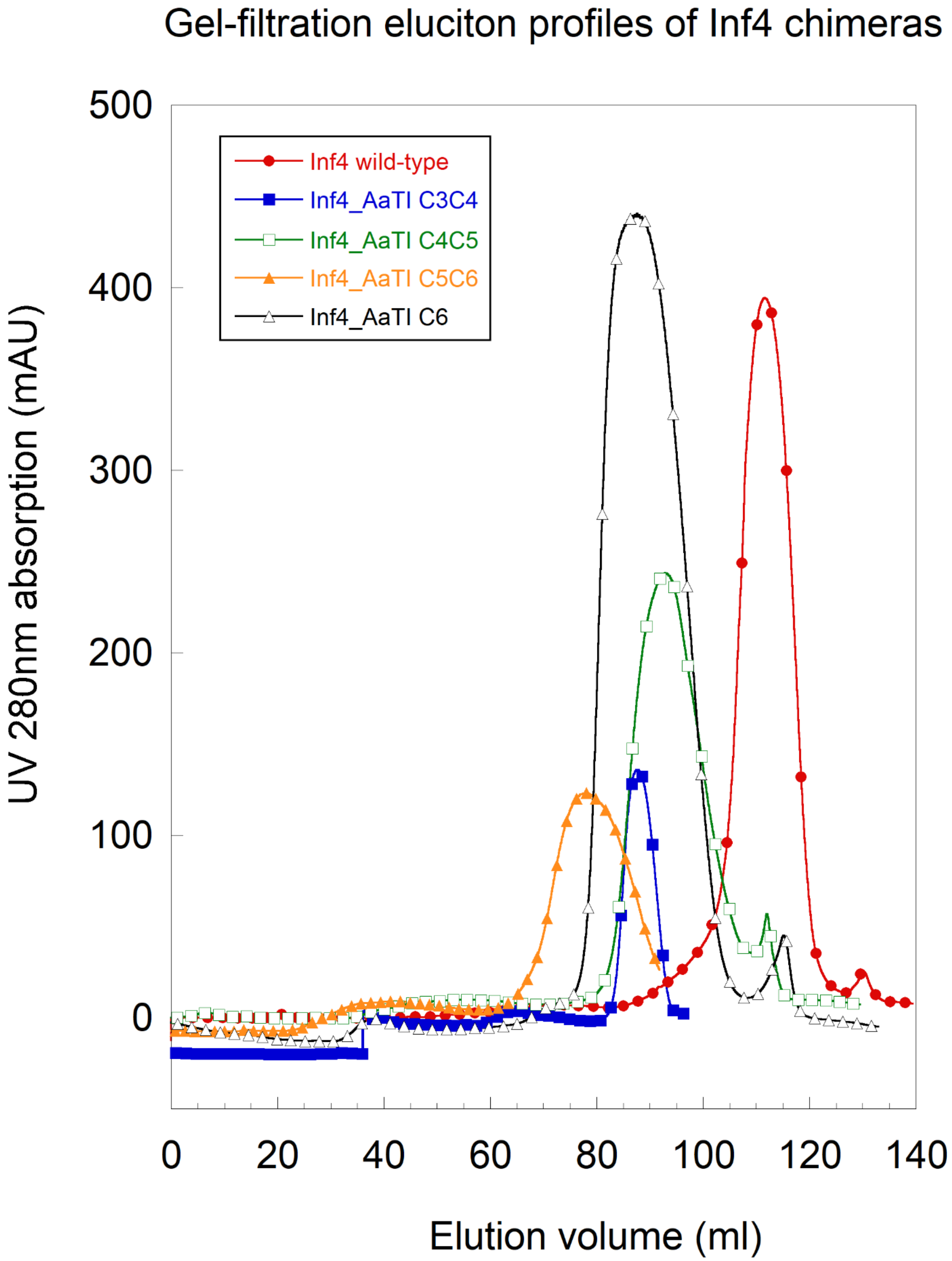

Gel-filtration eluciton profiles of Inf4 chimeras
Inf4 wild-type
Inf4_AaTI C3C4
Inf4_AaTI C4C5
Inf4_AaTI C5C6
Inf4_AaTI C6
UV 280nm absorption (mAU)
500
400
300
200
100
0
0
20
40
60
80
100
120
140
Elution volume (ml)

(B)

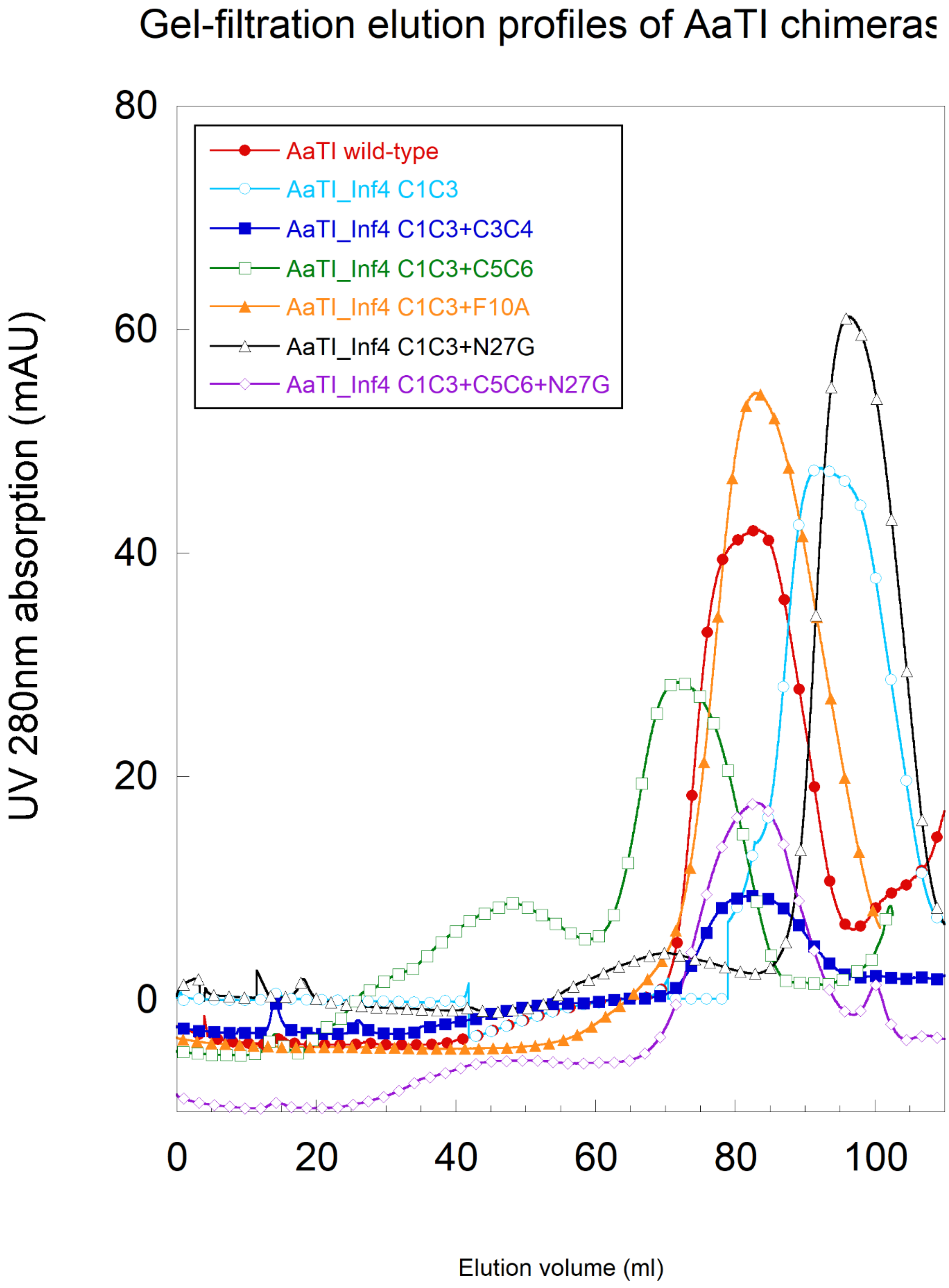

Gel-filtration elution profiles of AaTI chimeras
AaTI wild-type
AaTI_Inf4 C1C3
AaTI_Inf4 C1C3+C3C4
AaTI_Inf4 C1C3+C5C6
AaTI_Inf4 C1C3+F10A
AaTI_Inf4 C1C3+N27G
AaTI_Inf4 C1C3+C5C6+N27G
UV 280nm absorption (mAU)
80
60
40
20
0
0
20
40
60
80
100
Elution volume (ml)

Figure S1. Gel-filtration elution profiles of wild-type and chimeric Inf4 (A) and AaTI (B).

(A) Gel-filtration elution profiles of wild-type and chimeric proteins of Inf4: wild-type Inf4 (red, closed circles), Inf4_AaTI C3C4 (blue, closed squares), Inf4_AaTI C4C5 (green, open squares), Inf4_AaTI C5C6 (orange, closed triangles), and Inf4_AaTI C6 (black, open triangles). (B) Gel-filtration elution profiles of wild-type and chimeric proteins of AaTI: wild-type AaTI (red, closed circles), AaTI_Inf4 C1C3 (cyan, open circles), AaTI_Inf4 C1C3+C3C4 (blue, closed squares), AaTI_Inf4 C1C3+C5C6 (green, open squares), AaTI_Inf4 C1C3+F10A (orange, closed triangles), AaTI_Inf4 C1C3+N27G (black, open triangles), and AaTI_Inf4 C1C3+C5C6+N27G (violet, open rhombuses). Gel-filtration was performed using HiLoad 16/600 Superdex 75 pg columns (16 × 600 mm).

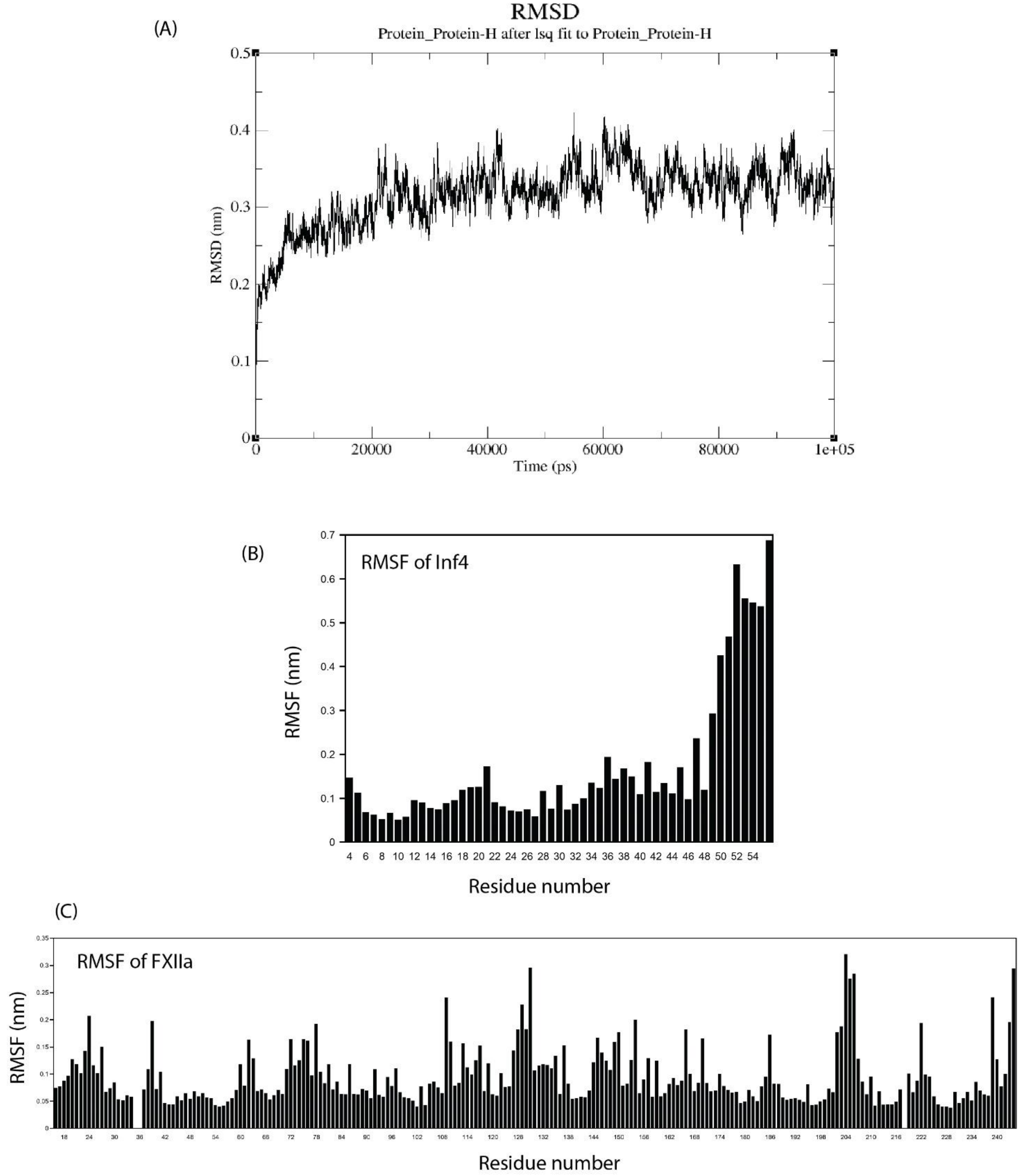

Figure S2. RMSD and RMSF plots of the Inf4:FXIIa complex from MD-simulation.

(A) RMSD plot, (B) RMSF plot of Inf4, and (C) RMSF plot of FXIIa of the Inf4:FXIIa complex obtained from MD simulation.

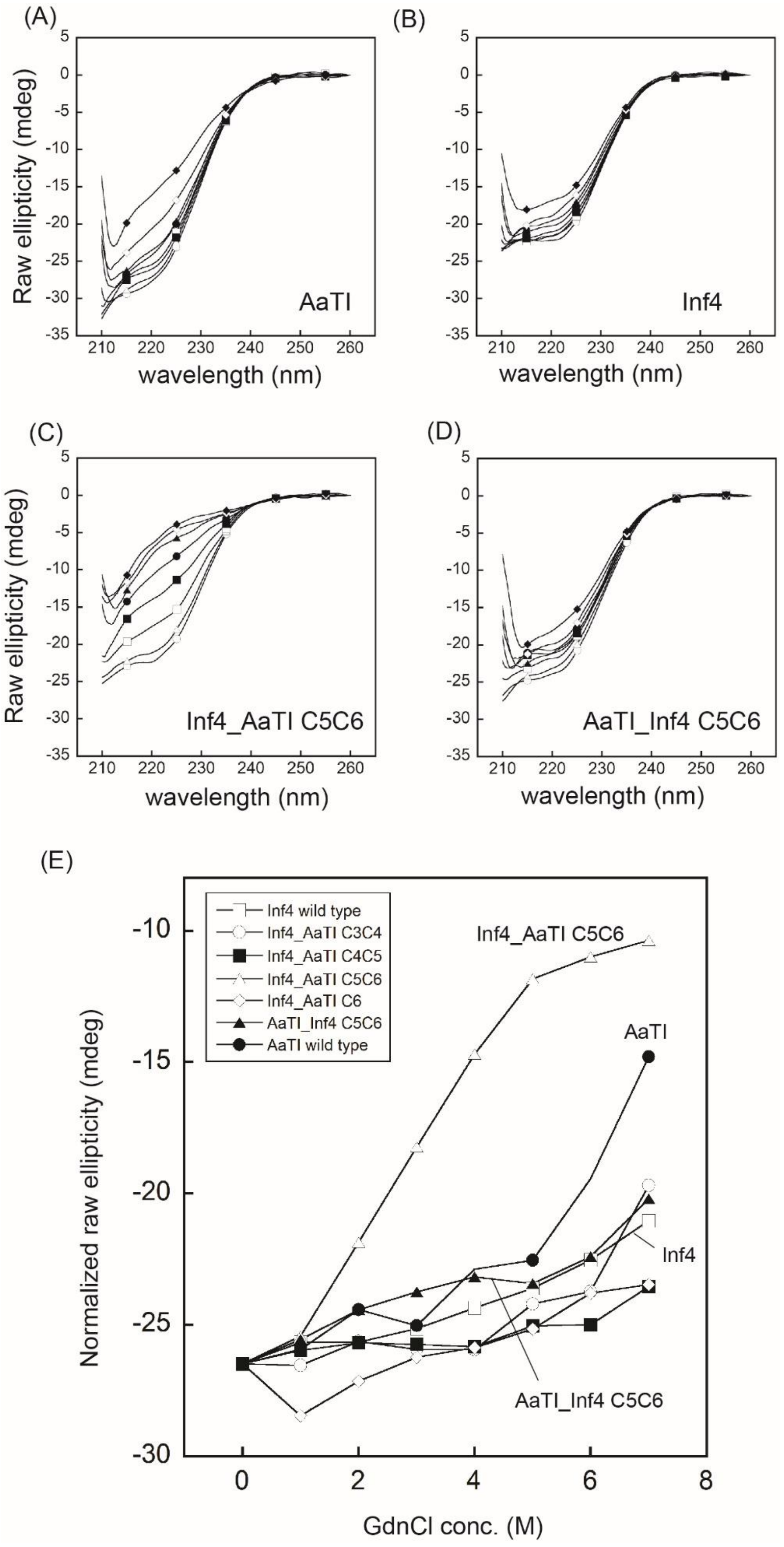
(A)
Raw ellipticity (mdeg)
wavelength (nm)
AaTI
(B)
wavelength (nm)
Inf4
(C)
Raw ellipticity (mdeg)
wavelength (nm)
Inf4_AaTI C5C6
(D)
wavelength (nm)
AaTI_Inf4 C5C6
(E)
Inf4 wild type
Inf4_AaTI C3C4
Inf4_AaTI C4C5
Inf4_AaTI C5C6
Inf4_AaTI C6
AaTI_Inf4 C5C6
AaTI wild type
Inf4_AaTI C5C6
AaTI
Inf4
AaTI_Inf4 C5C6
Normalized raw ellipticity (mdeg)
GdnCl conc. (M)

Figure S3. GdnCl denaturation (FarUV CD ellipticity at 222 nm) of Inf4 and AaTI chimera shows that the unstable scaffold of AaTI is caused by the C5C6 region.

The protein scaffold of AaTI is less stable due to the elongated C5C6 region**.** The stability of AaTI can be enhanced significantly by replacing the C5C6 region with that of Inf4 (AaTI_Inf4 C5C6). Alternatively, the stability of Inf4 can be reduced significantly by replacing the C5C6 region with that of AaTI (Inf4_AaTI C5C6). Replacement of other regions of Inf4 with those of AaTI does not change the stability significantly. (A)-(D) Far-UV CD spectra of wild-type AaTI, wild-type Inf4, Inf4_AaTI C5C6, and AaTI_Inf4 C5C6 in the presence of 0 M GdnCl (open circles), 1 M GdnCl (open triangles), 2 M GdnCl (open squares), 3 M GdnCl (closed squares), 4 M GdnCl (closed circles), 5 M GdnCl (closed triangles), 6 M GdnCl (open rhombuses), and 7 M GdnCl (closed rhombuses). Data are based on raw ellipticity (mdeg) values, and normalized to zero at 260 nm. (E) Plots showing GdnCl denaturation curves for wild-type AaTI (closed circles), wild-type Inf4 (open squares), Inf4_AaTI C5C6 (open triangles), AaTI_Inf4 C5C6 (closed triangles), Inf4_AaTI C3C4 (open circles), Inf4_AaTI C4C5 (closed squares), and Inf4_AaTI C6 (open rhombuses). Data are based on raw ellipticity (mdeg) values at 222 nm and normalized to wild-type AaTI at 0 M GdnCl.

```
                                                                              P14'
AaTI           -------------------ERGVCACPRIYMPVCGSNLKT----YNNDCLLRCEINSDLGR  38
Infestin4      -----------------EV-RNPCACFRNYVPVCGSDGKT----YGNPCMLNCAAQTKVP-  38
Infestin1      -----------------LE-ENDCACPRVLHRVCGSDGNT----YSNPCTLDCAKHEGKP-  38
LDTI           --------------------KKVCACPKILKPVCGSDGRT----YANSCIARCNGVSIKSE  37
BdellinKL      ----------------IN-ADTECVCTKELLRVCGEDGVT----YDNSCLATCHGTSVAHE  40
BdellinB3      --------------------DTECVCTKELHRVCGSDGVT----YDNECLATCHGASVAHD  37
PSTI           ---------DSLGREAKCYNELNGCTYEYRPVCGTDGDT----YPNECVLCFENRKRQT-  46
OMSPV3         --------LAAVSVDCSE-YPKPACTMEYRPLCGSDNKT----YGNKCNFCNAVVESNG-  46
CmpI-II        ---------------AEDC-VGRKACTREWYPVCGSDGVT----YSNPCNFSAQQEQCDP-  40
Rhodniin       -----------------EG-GEPCACPHALHRVCGSDGET----YSNPCTLNCAKFNGKP-  38
Dipetalin      -----------------FQ-GNPCECPRALHRVCGSDGNT----YSNPCMLTCAKHEGNP-  38
Greglin        SEDDGSASPESQEMSYTELPCPSICPLIYAPVCVEDSNQDFYLFVNECEVRKCGCEAGFV  60

AaTI           ----ANNLRKIADQACDNLTDNVNDFIPQEY-       65
Infestin4      ------GLKLVHEGRCQRSNVE---QF-----       56
Infestin1      ------DLVQVHEGPCDPNDHD---F------       55
LDTI           -------------GSCPTGI------------       44
BdellinKL      -------------HACEG--------------       45
BdellinB3      -------------HACEGHEEHHVDEHGEDHD       56
PSTI           ------SILIQKSGPC----------------       56
OMSPV3         ------TLTLSHFGKC----------------       56
CmpI-II        ------NITIAHMGEC----------------       50
Rhodniin       ------ELVKVHDGPCEPD-------------       51
Dipetalin      ------DLVQVHEGPCDEHDHD---F------       55
Greglin        YTFVPREMCKATTSLCPMQTKSS---------       83
```

Figure S4. Sequence alignment of Kazal-type inhibitors with AaTI showing residues at the P14’ position.

Sequence alignment of Kazal-type protease inhibitors showing the P14’ residues (boxed in red) in the C3C4 region. Most inhibitors contains a small or branched residue with a short side-chain (e.g., Gly, Ala or Ser); however, AaTI contains an Asn residue at this position.

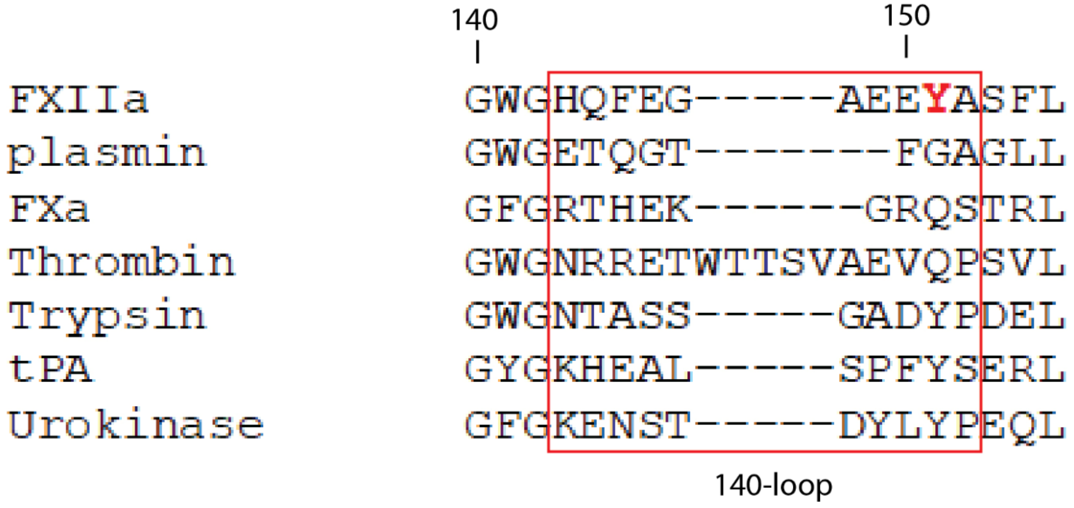

Figure S5. Sequence alignment of the 140-loop in selected serine proteases.

Sequence alignment of the 140-loop (boxed in red) from selected serine proteases using sequences, boundary of 140-loop based on Figure S1 in Dementiev *et al.*, 2018 [1]. The residue Y151 of FXIIa is bold-faced and in red.

References

1 Dementiev, A., Silva, A., Yee, C., Li, Z., Flavin, M. T., Sham, H. and Partridge, J. R. (2018) Structure of human plasma β-factor XIIa cocrystallized with potent inhibitors. Blood Advances. **2**, 549-557
